# Cases analysis and management strategies for ventricular shunt failure in hydrocephalus

**DOI:** 10.1097/MD.0000000000044425

**Published:** 2025-09-19

**Authors:** Jinfeng Zhang, Jiayong Lin, Yanhong Quan, Jiadi Zheng, Peng Zhang, Tao Jiang, Xiaoping Wang

**Affiliations:** a Department of Neurosurgery, Xiamen Hospital of Traditional Chinese Medicine, Xiamen, China; b Department of Neurosurgery, Fujian Sanbo Funeng Brain Hospital, Fuzhou, China; c Department of Neurology, Xiamen Hospital of Traditional Chinese Medicine, Xiamen, China.

**Keywords:** hydrocephalus, shunt failure, ventriculoatrial shunting

## Abstract

**Rationale:**

This report details 2 complex cases of recurrent ventriculoperitoneal (VP) shunt failure due to peritoneal pathology and infection, highlighting the clinical rationale for utilizing ventriculatrial (VA) shunting as a salvage procedure and the potential for subsequent VP shunt reimplantation after peritoneal recovery.

**Patient concerns:**

Case 1: A 57-year-old male presented with recurrent episodes of progressive gait instability, somnolence, cognitive decline, and vomiting over 9 months following initial VP shunt placement, despite multiple surgical revisions. Case 2: A 46-year-old male with a long history of shunt complications presented with worsening gait instability, impaired concentration, episodic dizziness, and intermittent confusion approximately 16 months after a VA shunt was placed as a secondary measure.

**Diagnoses:**

Both cases were diagnosed with recurrent hydrocephalus secondary to shunt failure. Case 1 was diagnosed with distal VP shunt obstruction caused by peritoneal adhesions, followed by a subsequent shunt infection confirmed by cerebrospinal fluid analysis. Case 2 was diagnosed with VA shunt valve failure despite correct catheter position, as indicated by shunt pressure monitoring and persistent symptoms.

**Interventions:**

Case 1: After multiple unsuccessful peritoneal catheter revisions and externalization for infection control, the patient was converted to a VA shunt. Case 2: Following the failure of VA shunt valve adjustments, abdominal ultrasound confirmed peritoneal recovery. The VA shunt was subsequently replaced with a contralateral VP shunt featuring a more granular adjustable valve.

**Outcomes:**

Case 1: Conversion to a VA shunt resulted in full neurological recovery, resolution of hydrocephalus symptoms, and no further complications at follow-up. Case 2: Contralateral VP shunt reimplantation led to the resolution of all neurological symptoms, significant functional recovery, and a return to independent daily activities, with no recurrence at the 6-month follow-up.

**Lessons:**

VA shunting is a safe and effective salvage procedure for patients with VP shunt failure attributable to peritoneal complications. Furthermore, these cases demonstrate that the peritoneal cavity can recover over time, allowing for successful VP shunt reimplantation if distal catheter failure recurs. A dynamic and individualized management strategy is essential for complex hydrocephalus cases.

## 1. Background

Hydrocephalus is a common neurological condition characterized by excessive accumulation of cerebrospinal fluid (CSF) in the brain, leading to increased intracranial pressure and associated neurological deficits. The management of hydrocephalus is complicated by systemic challenges, such as limited access to advanced imaging and surgical expertise, which can delay diagnosis and treatment of shunt failure.^[1]^ Ventriculoperitoneal (VP) shunting remains the standard treatment for hydrocephalus, providing long-term CSF diversion to the peritoneal cavity.^[2]^ However, VP shunt complications, particularly distal catheter obstruction and infection, continue to pose significant clinical challenges, often necessitating surgical revision.^[3,4]^

In cases where VP shunting fails due to peritoneal catheter dysfunction, such as mechanical occlusion or CSF malabsorption, alternative strategies, including ventriculoatrial (VA) shunting, may be considered.^[5,6]^ VA shunting, which diverts CSF into the right atrium of the heart, has been used as a secondary option for patients with peritoneal failure. While concerns about thromboembolic complications exist, VA shunting has proven to be a safe and effective alternative for select patients.

This study presents 2 unique cases of VP shunt failure due to peritoneal catheter obstruction and infection, highlighting the transition to VA shunting and the potential for VP shunt reinsertion after peritoneal recovery. These cases emphasize the dynamic nature of hydrocephalus management and the importance of individualized treatment approaches.

## 2. Ventriculoatrial shunt procedure

**Figure 1.**
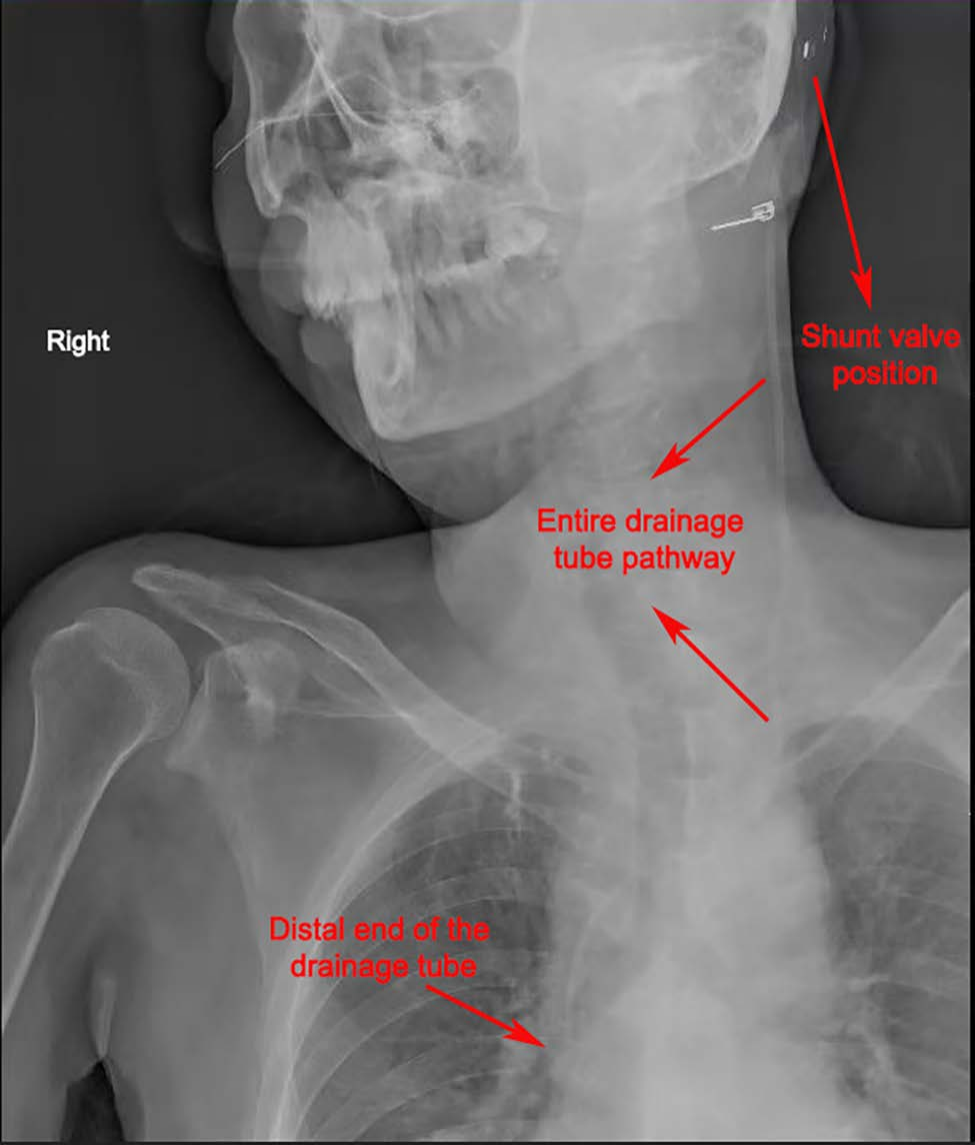
The shunt catheter pathway below the valve of the VA shunt. VA = ventriculoatrial.

The ventriculoatrial (VA) shunt procedure was performed under general anesthesia in a sterile operating room environment. The patient was placed in a supine position with the head slightly elevated to optimize venous access. The procedure involved the following steps (Fig. 1):

1. Incision and exposure: a small postauricular incision was made behind the ear to access the previously placed VP shunt valve. The distal peritoneal catheter was carefully dissected and removed.
2. Venous access and catheter insertion: the right internal jugular vein was identified using ultrasound guidance to minimize complications. A percutaneous puncture was performed, and an 8F introducer sheath was inserted to facilitate catheter placement. The shunt tubing was flushed with heparinized saline to prevent clot formation. Heparinized saline was used to flush the shunt tubing to prevent clot formation and ensure patency of the catheter during the procedure. This is especially important in the venous environment, where the risk of thrombus formation can be elevated due to the prolonged catheter presence.
3. Advancement of the catheter: under fluoroscopic guidance, the distal catheter was advanced through the internal jugular vein into the superior vena cava and further into the right atrium. Proper positioning at the junction of the superior vena cava and right atrium was confirmed using real-time imaging.
4. Connection to the proximal shunt system: the distal catheter was tunneled subcutaneously from the neck incision to the postauricular site and connected to the shunt valve. The connection was secured with nonabsorbable sutures to prevent dislodgement.
5. Closure and postoperative care: the incision was closed in layers, and a sterile dressing was applied. Postoperatively, the patient was closely monitored for hemodynamic stability, signs of infection, and proper shunt function. A chest X-ray was performed to confirm the final position of the catheter tip in the right atrium.

### 2.1. Postoperative management and follow-up

1. Patients were monitored in the intensive care unit for the first 24 hours for any signs of cardiac complications, including arrhythmias or thromboembolic events.
2. Serial imaging, including chest X-ray and echocardiography, was conducted to ensure proper catheter placement and function.
3. Patients were followed up at regular intervals with neurological assessments, shunt function tests, and routine imaging to detect potential complications such as catheter migration or shunt malfunction.
4. In cases of suspected shunt dysfunction, further evaluations, including pressure adjustments and shunt revision, were considered based on clinical presentation.

## 3. Cases presentation

### 3.1. Case 1

A 57-year-old male with a history of pituitary tumor resection on November 26, 2019, and ventriculoperitoneal (VP) shunt placement for hydrocephalus on July 17, 2020, was admitted on July 2, 2021, due to a 2-week history of progressive gait instability and somnolence. Upon admission, the patient exhibited marked unsteady gait, drowsiness, and cognitive sluggishness. A computed tomography (CT) scan of the brain demonstrated ventricular enlargement with evidence of increased intracranial pressure. The imaging findings included postoperative hydrocephalus, disappearance or narrowing of cerebral sulci, and significant compression of brain tissue. Given the patient’s clinical symptoms, the VP shunt valve was gradually adjusted from 1.5 to 0.5 settings to optimize CSF drainage. However, despite these adjustments, the patient’s symptoms did not improve.

**Figure 2.**
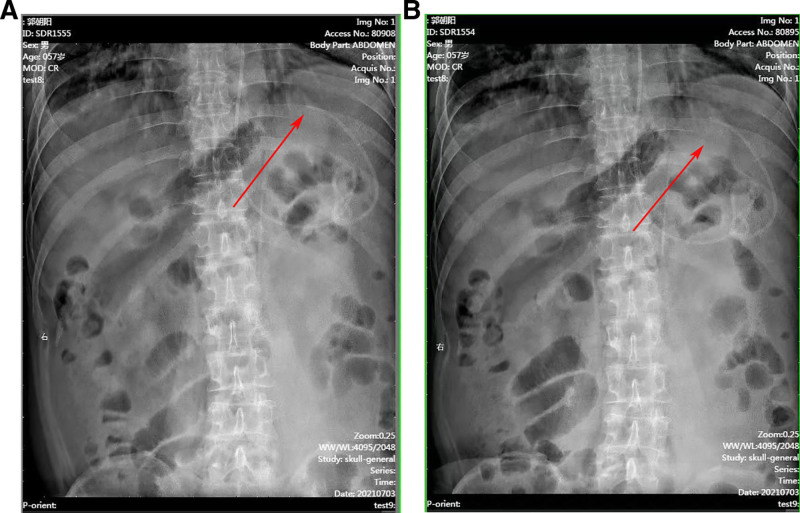
X-ray images of the peritoneal catheter position of a VP shunt at 2 different time points (A and B). The images show no change in the position of the peritoneal catheter between the 2 X-rays. Arrows indicate the peritoneal catheter tip position. VP = ventriculoperitoneal.

To investigate possible distal catheter dysfunction, 2 consecutive abdominal X-rays were performed 6 hours apart. The images revealed that the peritoneal catheter tip remained in the same position, raising suspicion of distal catheter blockage and impaired CSF absorption (Fig. 2A and B).

On July 6, 2021, surgical exploration confirmed adhesion and partial blockage of the peritoneal catheter. The catheter was repositioned into the right peritoneal cavity in an attempt to restore proper CSF drainage. Postoperatively, the patient’s symptoms improved temporarily, with a reduction in gait instability and somnolence.

Three months later on October 11, 2021, the patient presented again with dizziness, unsteady gait, and cognitive decline. A repeat CT scan suggested recurrent hydrocephalus with worsening ventricular dilation. Considering the patient’s previous catheter dysfunction, another peritoneal catheter revision was performed. Postoperatively, the patient showed mild improvement, but over the following months, he developed systemic malaise, and generalized weakness. Abdominal imaging revealed encapsulated peritoneal fluid collection surrounding the catheter tip, strongly suggesting peritoneal shunt failure. Laparoscopic examination (February 5, 2022) confirmed severe peritoneal adhesions and localized inflammation, which were likely responsible for poor CSF absorption. Given the significant peritoneal pathology, the catheter was relocated to the pelvic cavity to optimize CSF absorption.

**Figure 3.**
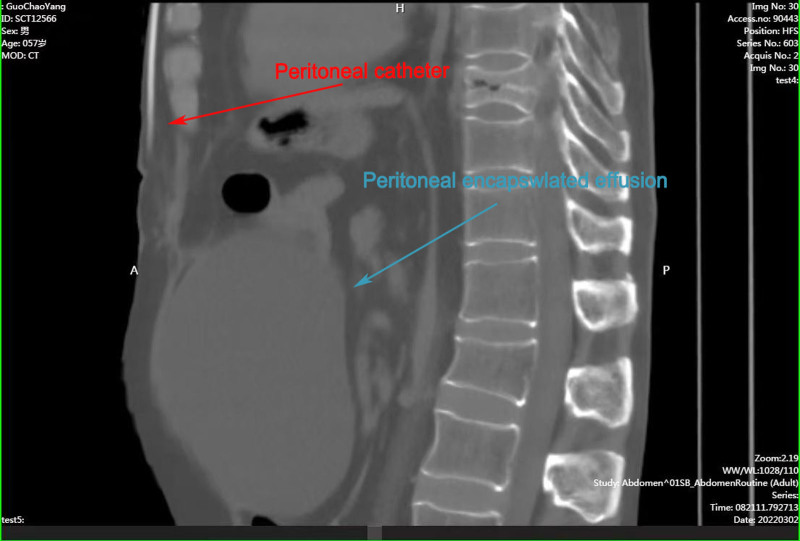
Abdominal CT showed the peritoneal catheter and peritoneal encapsulated effusion. The red arrow indicates the peritoneal catheter, while the blue arrow points to the peritoneal encapsulated effusion. CT = computed tomography.

Within weeks of the second revision, the patient developed progressive drowsiness and recurrent vomiting, raising concerns about worsening intracranial pressure and potential infection. On Feburary 28, 2022, abdominal imaging demonstrated persistent peritoneal fluid collection (Fig. 3), and cerebrospinal fluid (CSF) analysis showed elevated white blood cell counts and protein, indicating an active infection. Given the strong suspicion of shunt-related infection, the peritoneal end of the shunt was externalized for drainage. This intervention resulted in significant symptom relief, with improved mental status and resolution of vomiting.

CSF cultures remained negative after 2 weeks of empirical broad-spectrum antibiotic therapy. Once the infection was fully controlled, alternative CSF diversion was considered to prevent further peritoneal complications.

Due to recurrent peritoneal complications and the failure of VP shunting, the patient underwent ventriculoatrial (VA) shunt placement as a salvage procedure on April 29, 2022. The procedure was performed under fluoroscopic guidance to ensure proper catheter placement within the right atrium. Postoperatively, the patient exhibited full neurological recovery with resolution of hydrocephalus symptoms. Follow-up imaging confirmed appropriate CSF diversion and stable ventricular size. The patient remained symptom-free during subsequent clinical follow-up, with no further episodes of catheter malfunction or infection. The timeline is showed in Table 1.

**Table 1:** Case 1: recurrent hydrocephalus with VP shunt failure and conversion to VA shunt.

Date	Event	Intervention/outcome
November 26, 2019	Diagnosis of pituitary tumor	“Classic sellar tumor resection + nerve decompression”; pathology confirmed pituitary adenoma
July 17, 2020	New-onset hydrocephalus (symptoms: drowsiness)	VP shunt placement (medtronic valve, initial setting: 1.5). Improved consciousness post-op
July 2, 2021	Recurrent symptoms (gait instability); CT: ventriculomegaly	Valve adjusted to 0.5; imaging confirmed distal obstruction due to adhesions
July 6, 2021	First distal revision	Catheter repositioned to right abdomen. Symptoms resolved
October 11, 2021	Recurrence (dizziness, gait disturbance); CT: shunt failure	Second distal revision. Improved after 1 wk
February 5, 2022	Third recurrence; laparoscopy confirmed adhesions	Adhesiolysis + catheter repositioning to pelvis. Temporary relief
February 28, 2022	Infection (CSF analysis+) + encapsulated abdominal fluid	Emergency distal externalization. Infection controlled
April 29, 2022	CSF normalization	Converted to VA shunt (valve set to 1.0). Long-term symptom resolution

CSF = cerebrospinal fluid, CT = computed tomography, VA = ventriculoatrial, VP = ventriculoperitoneal.

## 3.1.1. Patient’s perspective. 

After undergoing the VA shunt procedure, the patient reported a significant improvement in symptoms. He felt more energetic overall. The patient expressed satisfaction with the outcome, noting that the transition to VA shunting provided much-needed relief. He also commented that the absence of major complications post-surgery further contributed to his sense of recovery and well-being.

### 3.2. Case 2

A 46-year-old male with a history of multiple hydrocephalus-related surgeries, including ventriculoperitoneal (VP) shunt placement, was admitted on January 3, 2024, with progressive cognitive decline, intermittent confusion, and occasional episodes of unconsciousness over the past month. The patient had undergone multiple previous interventions for hydrocephalus, and his condition had been complicated by repeated infections, catheter dysfunction, and shunt malfunctions over the years.

**Figure 4.**
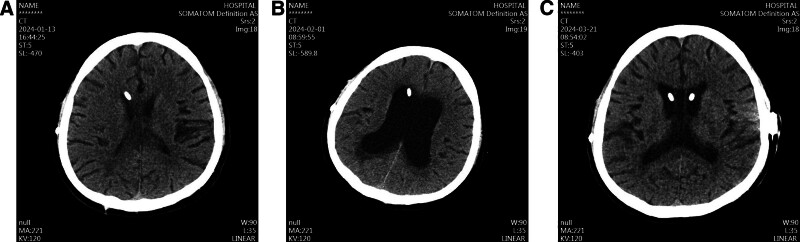
Representative CT scan images. (A) (January 13, 2024): This CT scan shows slit-like ventricles, indicating over-drainage. (B) (February 1, 2024): This CT scan shows significant ventricular enlargement, suggesting under-drainage. (C) (March 21, 2024): This CT scan, taken postoperatively after right-sided ventriculoperitoneal shunt surgery, shows normal ventricular morphology with appropriate drainage. CT = computed tomography.

The patient had previously underwent 4th ventricular tumor resection on May 19, 2017, which resulted in obstructive hydrocephalus, necessitating VP shunt placement on May 25, 2017. Over the following years, he suffered multiple VP shunt complications, including CSF infections and persistent CSF leakage. In response, he underwent removal of the Ommaya reservoir and temporary external ventricular drainage to manage the infection on July 1, 2022. After infection resolution, a ventriculoatrial (VA) shunt was implanted as an alternative CSF diversion method on September 15, 2022 (Fig. 4).

Approximately 16 months after the VA shunt placement, the patient developed worsening gait instability, impaired concentration, and episodic dizziness. Given his complex history, brain imaging was performed and showed ventricular dilation indicating possible shunt dysfunction. In response, a stepwise adjustment of the VA shunt pressure valve (from 1.5–0.5 settings) was attempted, but his symptoms failed to improve. Further investigation into the VA shunt function was undertaken. Echocardiography and chest radiography confirmed that the VA catheter was correctly positioned within the right atrium. However, shunt pressure monitoring tests suggested valve failure, leading to inadequate CSF diversion.

Given his history of peritoneal catheter dysfunction, an abdominal ultrasound was performed to assess the viability of the peritoneal cavity for potential VP shunt reimplantation. The ultrasound revealed no residual adhesions or fluid collections, suggesting that the peritoneal cavity had recovered sufficiently to support a new VP shunt. Based on these findings, a decision was made to proceed with contralateral VP shunt placement.

A new VP shunt was implanted on the contralateral side on March 11, 2023, incorporating an more granular adjustable valve to ensure optimal CSF flow regulation. Intraoperatively, adequate CSF flow through the newly placed VP shunt system was confirmed. There were no signs of postoperative complications, and the peritoneal cavity demonstrated good CSF absorption.

Following the contralateral VP shunt placement, the patient exhibited marked clinical improvement. His gait instability, cognitive deficits, and dizziness gradually resolved, and his overall neurological function improved significantly. Follow-up brain imaging confirmed successful CSF diversion, with stable ventricular size and no evidence of shunt obstruction, infection, or catheter migration. At the 6-month follow-up visit, the patient remained neurologically stable, without any recurrence of hydrocephalus-related symptoms. He returned to independent daily activities and demonstrated significant functional recovery. The timeline is showed in Table 2.

**Table 2:** Case 2: complex VP shunt failure with infection and dual-system strategy.

Date	Procedure/intervention	Outcome/symptom changes
May 19, 2017	Right ventricular occipital puncture + Ommaya reservoir placement + Suboccipital midline approach cerebellar vermis tumor resection	Relieved obstructive hydrocephalus; tumor resected
May 25, 2017	Right VP shunt placement	Hydrocephalus completely resolved
July 1, 2022	Right Ommaya reservoir removal + VP shunt externalization (abdominal end)	Removed source of intracranial and abdominal infection
September 15, 2022	Right VA shunt placement + removal of previous VP shunt system	Infection controlled; symptoms improved after VA shunt
January 3, 2024	Transferred to our hospital; repeated shunt valve adjustments	Unstable CSF drainage (under- or over-drainage); symptoms persisted
March 11, 2024	Abdominal ultrasound, contralateral placement of a new VP shunt with finer pressure adjustment	No adhesions/effusion; abdominal function recovered, supporting new VP shunt; symptoms significantly improved; no complications

CSF = cerebrospinal fluid, VA = ventriculoatrial, VP = ventriculoperitoneal.

## 3.2.1. Patient’s perspective.

Post-surgery, the patient was able to walk without assistance and regained independence in daily activities. *The patient is largely independent in daily activities* During follow-up visits, the patient showed clear consciousness and was able to express their thoughts and needs effectively. He described feeling “like myself again” and was very pleased with the restoration of his quality of life, expressing gratitude for the successful outcome of the surgery.

## 4. Discussion

Ventriculoperitoneal (VP) shunting is the most commonly performed procedure for the management of hydrocephalus. However, shunt dysfunction due to distal catheter obstruction and infection remains a major challenge, often necessitating surgical revisions.^[7,8]^ The complications associated with VP shunting significantly impact patient outcomes, increasing the risk of neurological deterioration and repeated hospitalizations. While advancements in shunt technology have improved longevity, mechanical failures and infection rates remain substantial concerns.^[9,10]^ In addition to technical challenges in shunt management, inequities in access to advanced neurosurgical care, particularly in underserved populations, can complicate timely diagnosis and treatment of shunt failure.^[11]^ External factors, such as disruptions in healthcare access during the COVID-19 pandemic, may exacerbate challenges in timely diagnosis and management of shunt failure, particularly in resource-limited regions.^[12]^

In both cases presented, abdominal X-rays were instrumental in diagnosing peritoneal catheter obstruction, as neither showed catheter displacement.^[13]^ However, X-ray serves only as an indirect sign of catheter dysfunction and should be combined with CT or MRI imaging to confirm the presence of hydrocephalus and increased pressure during shunt valve adjustment. This approach enhances diagnostic reliability for peritoneal catheter obstruction. Given the complexity of VP shunt dysfunction, additional imaging methods such as ultrasound and CT may help differentiate mechanical blockages from functional failures due to CSF absorption issues.^[14,15]^ The findings of this study suggest that peritoneal catheter blockage can often be relieved through catheter repositioning, providing temporary symptom relief while allowing further evaluation and management. However, the recurrence of catheter dysfunction in both cases suggests that repositioning alone may not always be a definitive solution, and alternative CSF diversion methods should be considered in patients with persistent peritoneal issues.^[16]^

One of the key aspects of hydrocephalus management is the prompt recognition and treatment of shunt infections. In both cases, peritoneal catheter externalization resulted in rapid symptom relief and infection control, which aligns with established protocols advocating external drainage as the first-line approach for VP shunt infections.^[17,18]^ The effectiveness of this strategy in preventing further complications and allowing infection-free reinsertion of the shunt is well documented in the literature. Despite its effectiveness, externalization requires prolonged inpatient care, raising concerns regarding hospital-acquired infections and extended recovery times. In cases where infection is recurrent, long-term strategies such as staged shunt replacement with antimicrobial-impregnated catheters may offer additional benefits.^[19]^

Ventriculoatrial (VA) shunting serves as a viable alternative for patients with peritoneal failure. Historically, VA shunts were associated with higher risks of complications such as thromboembolism, catheter-induced endocarditis, and systemic infections. However, advancements in surgical techniques, including the use of fluoroscopic guidance for precise catheter placement, and improvements in shunt materials have significantly improved outcomes, making VA shunting a reliable option when VP shunting is not feasible.^[13]^ The first case demonstrates that transitioning from VP to VA shunting can lead to complete resolution of symptoms without additional complications. Given the lower risk profile of modern VA shunts, this approach should be considered earlier in the management algorithm for patients with recurrent peritoneal complications.^[20]^ However, despite these advancements, there are still potential complications that must be considered. In addition to thromboembolism and endocarditis, pulmonary hypertension is a known risk due to increased venous pressure from the shunt’s drainage pathway. Over time, this increased pressure can strain the right side of the heart, potentially leading to right heart failure or other cardiovascular issues. Catheter migration is another complication, where the catheter may shift from its intended position in the right atrium. This migration can cause blockage, inadequate drainage, or even perforation of the heart or other structures. Regular imaging follow-up is essential to monitor for these potential complications. External factors, such as disruptions in healthcare access during the COVID-19 pandemic, may exacerbate challenges in timely diagnosis and management of shunt failure, particularly in resource-limited regions.^[21]^

The second case highlights the potential for VP shunt reinsertion following peritoneal recovery. The ability of the peritoneal cavity to self-repair and accommodate a shunt after a period of diversion or externalization has been previously suggested but is not routinely reevaluated in clinical practice. Several physiological mechanisms may contribute to this recovery, including the resolution of peritoneal inflammation, resorption of peritoneal fluid collections, and reepithelialization of mesothelial cells that line the peritoneal cavity.^[13,14]^ Inflammation-induced adhesions may also undergo partial remodeling or spontaneous regression over time, especially in the absence of persistent infection or mechanical irritation.^[15,16]^ In this patient, follow-up abdominal ultrasound revealed no residual adhesions or effusions, suggesting that the peritoneal environment had sufficiently normalized to support renewed CSF absorption. This clinical course supports the notion that VP shunting should not be permanently contraindicated in patients with prior peritoneal dysfunction. In selected patients, reassessment of peritoneal viability using imaging and functional evaluation may enable safe VP shunt reimplantation, potentially avoiding long-term VA shunting and its associated complications. A dynamic, individualized evaluation of peritoneal recovery may thus expand future options in complex hydrocephalus management. The first case in this series demonstrates that transitioning from VP to VA shunting can lead to complete resolution of symptoms without the development of additional complications. This reinforces the effectiveness of VA shunting as a treatment for patients with peritoneal failure who are not candidates for VP shunting. Given the lower risk profile of modern VA shunt systems, this approach should be considered earlier in the management algorithm for patients with recurrent peritoneal complication**s.** By adopting VA shunting sooner, patients may benefit from reduced surgical interventions and improved outcomes, especially in cases of repeated VP shunt failure.

These cases underscore the importance of individualized treatment strategies in managing hydrocephalus. A stepwise approach, including serial imaging, staged externalization, alternative shunt placement, and reassessment for VP shunt reinsertion, may provide optimal long-term outcomes for patients with complex shunt dysfunction. Additionally, multidisciplinary collaboration among neurosurgeons, infectious disease specialists, and radiologists is crucial to optimizing patient outcomes. Further prospective studies are needed to establish standardized protocols for decision-making in these challenging scenarios. The continued evolution of CSF diversion techniques, including minimally invasive endoscopic procedures and bioengineered shunt systems, may further refine treatment strategies in the future.^[22,23]^ Beyond current CSF diversion techniques, emerging therapies such as stem cell treatments for neurological disorders may offer novel approaches to address underlying brain pathology in hydrocephalus, particularly in cases with cognitive decline.^[24,25]^

These 2 cases of recurrent VP shunt failure provide critical insights for clinical decision-making in complex hydrocephalus management. The decision to convert to VA shunting should be guided by a triad of assessments: peritoneal status evaluation, where CT-documented extensive adhesions (Case 1) mandated VA conversion while ultrasonographic confirmation of peritoneal recovery (Case 2) permitted successful VP revision; infection control parameters, with both cases demonstrating the necessity of a minimum 2-week CSF normalization period prior to definitive shunt surgery; and patient-specific factors including age, comorbidities, and CSF characteristics. Notably, Case 2 challenges the conventional paradigm of permanent peritoneal failure by demonstrating that a 3 to 6 month observation period with serial imaging may identify candidates for VP shunt preservation. However, early VA conversion remains imperative for patients with recurrent obstructions (≥2 failures) or radiologically confirmed irreversible peritoneal pathology, provided comprehensive cardiac evaluation is performed preoperatively. While derived from specific clinical scenarios, these findings contribute significantly to the development of more individualized cerebrospinal fluid diversion algorithms.

## 5. Conclusion

This study suggests that serial abdominal X-rays performed 6 to 8 hours apart can be a useful tool for diagnosing VP shunt peritoneal catheter obstruction, though further studies are needed to establish its reliability. Temporary externalization of the shunt effectively alleviates hydrocephalus symptoms and accelerates infection resolution, allowing for safe reinsertion. VA shunting remains a practical alternative for patients with peritoneal failure, and in selected cases, peritoneal self-repair may permit the successful reimplantation of a VP shunt. These findings contribute to the evolving strategies for hydrocephalus management and highlight the need for a tailored approach based on individual patient conditions. However, this study has several limitations. The sample size is relatively small, which limits the generalizability of the findings. Additionally, the lack of long-term follow-up data makes it difficult to assess the durability of the observed outcomes. There may also be a potential selection bias in the decision to opt for VA shunting in certain patients, which could impact the overall conclusions. These factors warrant further investigation in larger, long-term studies to better understand the optimal strategies for managing complex hydrocephalus.

## Author contributions

**Conceptualization:** Tao Jiang, Xiaoping Wang.

**Data curation:** Jinfeng Zhang, Jiayong Lin, Yanhong Quan, Jiadi Zheng, Peng Zhang.

**Formal analysis:** Jinfeng Zhang, Jiayong Lin.

**Funding acquisition:** Tao Jiang, Xiaoping Wang.

**Investigation:** Jinfeng Zhang, Jiayong Lin, Yanhong Quan, Jiadi Zheng, Peng Zhang.

**Methodology:** Jinfeng Zhang, Jiayong Lin, Yanhong Quan, Jiadi Zheng, Peng Zhang.

**Project administration:** Tao Jiang, Xiaoping Wang.

**Resources:** Jinfeng Zhang, Jiayong Lin, Yanhong Quan, Jiadi Zheng, Peng Zhang.

**Software:** Jinfeng Zhang, Jiayong Lin, Yanhong Quan, Jiadi Zheng, Peng Zhang.

**Supervision:** Tao Jiang, Xiaoping Wang.

**Validation:** Jinfeng Zhang, Jiayong Lin, Yanhong Quan, Jiadi Zheng, Peng Zhang.

**Visualization:** Jinfeng Zhang, Jiayong Lin.

**Writing – original draft:** Jinfeng Zhang.

**Writing – review & editing:** Tao Jiang, Xiaoping Wang.
